# Composition and changes of blood microbiota in adult patients with community-acquired sepsis: A *pilot* study from bench to bedside

**DOI:** 10.3389/fcimb.2022.1067476

**Published:** 2022-12-13

**Authors:** Bálint Gergely Szabó, Rebeka Kiss, Nóra Makra, Kinga Pénzes, Eszter Vad, Katalin Kamotsay, Dóra Szabó, Eszter Ostorházi

**Affiliations:** ^1^ South Pest Central Hospital, National Institute of Hematology and Infectious Diseases, Budapest, Hungary; ^2^ Departmental Group of Infectious Diseases, Department of Haematology and Internal Medicine, Semmelweis University, Budapest, Hungary; ^3^ Institute of Medical Microbiology, Semmelweis University, Budapest, Hungary

**Keywords:** sepsis, blood microbiota, blood culture, 16S rRNA, community-acquired sepsis

## Abstract

**Background:**

Characteristics of the blood microbiota among adult patients with community-acquired sepsis are poorly understood. Our aim was to analyze the composition of blood microbiota in adult patients with community-acquired sepsis, and correlate changes with non-septic control patients.

**Methods:**

A prospective observational study was carried out by including adult patients hospitalized for community-acquired sepsis at our center between January and November 2019, by random selection from a pool of eligible patients. Study inclusion was done on the day of sepsis diagnosis. Community acquisition was ascertained by *a priori* exclusion criteria; sepsis was defined according to the SEPSIS-3 definitions. Each included patient was matched with non-septic control patients by age and gender in a 1:1 fashion enrolled from the general population. Conventional culturing with BacT/ALERT system and 16S rRNA microbiota analysis were performed from blood samples taken in a same time from a patient. Abundance data was analyzed by the *CosmosID HUB* Microbiome software.

**Results:**

Altogether, 13 hospitalized patients were included, 6/13 (46.2%) with sepsis and 7/13 (53.8%) with septic shock at diagnosis. The most prevalent etiopathogen isolated from blood cultures was *Escherichia coli*, patients mostly had intraabdominal septic source. At day 28, *all-cause* mortality was 15.4% (2/13). Compared to non-septic control patients, a relative scarcity of *Faecalibacterium*, *Blautia, Coprococcus* and *Roseburia* genera, with an abundance of *Enhydrobacter*, *Pseudomonas* and *Micrococcus* genera was observed among septic patients. Relative differences between septic vs. non-septic patients were more obvious at the phylum level, mainly driven by *Firmicutes* (25.7% vs. 63.1%; p<0.01) and *Proteobacteria* (36.9% vs. 16.6%; p<0.01). The alpha diversity, quantified by the *Chao1* index showed statistically significant difference between septic vs. non-septic patients (126 ± 51 vs. 66 ± 26; p<0.01). The Bray-Curtis beta diversity, reported by principal coordinate analysis of total hit frequencies, revealed 2 potentially separate clusters among septic vs. non-septic patients.

**Conclusion:**

In adult patients with community-acquired sepsis, specific changes in the composition and abundance of blood microbiota could be detected by 16S rRNA metagenome sequencing, compared to non-septic control patients. Traditional blood culture results only partially correlate with microbiota test results.

## 1 Introduction

Sepsis is a dysregulated host immune response arising from an infection, which may lead to multi-systemic inflammation, organ failure and ultimately, patient death (Singer et al., 2016). According to recent data from the *Centers for Disease Control and Prevention*, as high as 87% of sepsis present before the patient is admitted to a healthcare facility, pointing to a significant community-level acquision in a relevant proportion of all cases (CDC, 2022). By most literature sources, community-acquired sepsis is defined as a case of sepsis with any severity (given by the either ACCP/SCCM SIRS based or the SEPSIS-3 consensus criteria), and a clinical onset between 48 to 72 hours after hospital admission, in a patient without any recent connection to healthcare facilities or nosocomial activities (Szabo et al., 2019).

According to the literature, the spectrum of etiopathogens most frequently isolated from clinical cases of community-acquired sepsis in immunocompetent adults is similar across different studies: the main isolated organism is *Escherichia coli*, followed by *Staphylococcus aureus*, *Streptococcus pneumoniae* and *Klebsiella pneumoniae*, lastly other *Streptococci* and Gram negative bacteria. However, by utilizing commercially available classical culturing or non-culture based microbiological methods (eg. antigen detection, serology or polymerase chain reaction [PCR] assays), an etiology could only be documented in 60–75% of all cases, even with concomitant bacteremia rates of community-acquired sepsis falling between 15 and 35% (Goncalves-Pereira et al., 2013; Sogaard et al., 2015; Henriksen et al., 2017). These limitations might affect clinical risk stratification and antimicrobial stewardship efforts of targeted de-escalation.

In recent years, the existence of an “eubiotic” blood microbiota has been proposed in healthy human individuals by the literature. One review assessed that its three chief components, detected mainly by 16s rRNA gene sequencing at the phyla level, were *Proteobacteria*, *Actinobacteria* and *Firmicutes*, as they contributed to >70% of the blood microbiota, while *Bacteroidetes*, *Fusobacteria*, *Cyanobacteria*, *Verrucomicrobia* and *Acidobacteria* only constitute the minority. In contrast, *Firmicutes* and *Bacteroidetes* are the typical phyla of the human gut microbiome (Suparan et al., 2022). Despite encouraging results, another review found that the main limitation to properly interpret the phylogenetic diversity of the healthy human blood microbiota lies in significant inter-research variability (Castillo et al., 2019). In septic patients, a “dysbiotic” shift of blood microbiota has been observed by some authors, with a higher ratio of the phyla *Proteobacteria* and *Bacteroidetes*, along with a reduction in *Actinobacteria* and a rise in *Agrococcus* (Paisse et al., 2016; Gosiewski et al., 2017). However, characteristic changes of the blood microbiota abundance among adults with community-acquired sepsis are still poorly understood, despite the vast rates of this clinical scenario among all documented septic cases, and needs further evaluation. Therefore, our aim was to assess the composition and changes of blood microbiota in adult patients with community-acquired sepsis, and correlate these changes to non-septic control patients.

## 2 Materials and methods

### 2.1 Study design, patient enrollment and patient–control matching

A prospective observational study was carried out by including patients from a cohort of eligible patients by computerized random selection on the day of sepsis diagnosis ascertainment. All consecutive adult (≥18 years of age at diagnosis) patients hospitalized for community-acquired sepsis at the study site between January and November 2019 were eligible for inclusion. The study site is a national-level referral center with >200 ward and intensive care unit beds for infectious and malignant hematological diseases. The study was in accordance with the Helsinki Declaration and national ethical standards. The study protocol was approved by the Institutional Review Board of Semmelweis University (Permission Number: SE RKEB: 87/2019) and South Pest Central Hospital, National Institute of Hematology and Infectious Diseases (Permission Number: ESZSZK EB 37/2016). Written informed consent was obtained from each included patient.

Sepsis was defined according to the SEPSIS-3 consensus definitions (Singer et al., 2016). Community acquisition was ascertained if sepsis onset or diagnosis was within ≤72 h from hospital admission or transportation occurred to the study site from the community or another hospital within the time frame, plus by applying *a priori* exclusion criteria, detailed *in extenso* in an earlier publication of our group (Szabo et al., 2019). Briefly, patients with nosocomial infections of any kind, recent hospitalizations, regular outpatient visits to clinics or long-term care facility residence were excluded. Each included septic patient was matched by age and gender (or best available option) with a non-septic control patient in a 1:1 ratio, enrolled from the general non-hospitalized population.

### 2.2 Data collection, follow-up and outcomes

Patient-level data were anonymously transferred to a standardized electronic case report form by manual extraction from healthcare charts. Data collected were: 1) age and gender, 2) comorbidities, 3) clinical presentation (symptom duration before hospitalization, severity and source of sepsis, length of hospitalization), 4) laboratory results (peripheral white blood cell count, absolute neutrophil granulocyte and lymphocyte counts, serum C-reactive protein [CRP] and procalcitonin [PCT] levels), 5) microbiological results (blood culture and 16S rRNA microbiota analyzes), 6) types and durations of empirical and targeted antimicrobial therapies, 7) clinical outcomes.

At the study site, diagnostic and therapeutic approach of septic patients was facilitated by an annually updated *in-house* written protocol since 2016. Patients were followed up for a total of 28 days, a post-discharge follow-up was done by telephone, e-mails and cross-checking in the National eHealth Infrastructure (social security database of Hungary). Clinical outcomes were *all-cause* mortality and need for intensive care unit (ICU) admission. Baseline variables were collected on the day of sepsis diagnosis, microbiological data and clinical outcomes were ascertained at 28 days post-admission.

### 2.3 Traditional blood culture method

From each participant, up to 10 mL per bottle of venous whole blood was inoculated into BacT/ALERT FA (bioMérieux, Marcy-l’Étoile, France) culture bottles for the cultivation of aerobic bacteria and fungi (2 bottles per patient), and another 10 mL of whole blood was inoculated into FN bottles for the cultivation of anaerobic bacteria promptly after sepsis diagnosis (2 bottles per patient). The skin site was mechanically cleaned with soap, and disinfected with 10% povidone-iodine and 2% chlorhexidine digluconate + 95% ethyl alcohol solutions three times before venipuncture. Until transportation to the laboratory, inoculated bottles were stored at +24°C. At the laboratory, these were then placed in an automated microbial detection system at +37°C (BACT/ALERT 3D, bioMérieux, Marcy-l’Étoile, France). After a positive signal of potential growth was detected, a Gram stained smear was prepared and aerobic, anaerobic and fungal cultivation began on the appropriate solid media. Negative blood culture results were interpreted on the basis of negative microscopic and culture procedures, after incubation of the bottles for 3 weeks. Identification of an organisms isolated from a positive blood culture was accomplished by matrix-assisted laser desorption/ionization time-of-flight mass spectrometry (MALDI/TOF–MS). Antibiotic susceptibility testing of isolates was carried out according to the recommendations given by the European Committee on Antimicrobial Susceptibility Testing (EUCAST). Apart from blood cultures, other relevant clinical samples were also collected from patients for non-culture based methods (e.g. *in vitro* urinary antigen detection for *S. pneumoniae* and *L. pneumophila*, *in vitro* toxin detection for *C. difficile* etc.), as determined by the clinical context at the discretion of the attending physician. A pathogen was accepted as a causative organism of sepsis if it was microbiologically identified from a clinically relevant sample during a compatible case presentation. The traditional blood culture and non-culture based methods were carried out at the Core Microbiology Laboratory of South Pest Central Hospital, National Institute of Hematology and Infectious Diseases (Budapest, Hungary).

### 2.4 16S rRNA microbiota analysis

From each patient, at least 3-3 mLs of whole blood were collected into citrate filled VACUETTE collection tubes (Greiner Bio-One, Stonehouse, UK), and after tube rotation, immediately frozen to –80°C. Skin site preparation was performed as presented earlier. DNA isolation was performed by NucleoSpin Blood, Mini kit (Macherey-Nagel, Allentown, PA, USA) according to the instructions of the manufacturer. Concentration of genomic DNA was measured using a Qubit2.0 Fluorometer with Qubit dsDNA HS Assay Kit (Thermo Fisher Scientific, Waltham, MA, USA). Bacterial DNA was amplified with tagged primers covering the V3-V4 region of the bacterial 16S rRNA gene. PCR and DNA purifications were performed according to Illumina’s protocol. PCR product libraries were assessed using DNA 1000 Kit with Agilent 2100 Bioanalyzer (Agilent Technologies, Waldbronn, Germany). Equimolar concentrations of libraries were pooled and sequenced on Illumina MiSeq platform (Illumina, San Diego, CA, USA) using MiSeq Reagent Kit v3 (600 cycles PE). To ensure reproducibility, each blood sample was independently extracted. To avoid false results (e.g. contamination) and to increase the reliability of the study, all analysis procedures were done in duplicate from 2 different isolated DNA samples of each donor. In order to evaluate the contribution of extraneous DNA from reagents, extraction negative controls and PCR negative controls were included in every run. Raw sequencing data were retrieved from the Illumina BaseSpace and the data were analyzed by the CosmosID bioinformatics platform (Yan et al., 2019), (CosmosId, 2022). The 16S rRNA metagenome sequencing was carried out at the Institute of Medical Microbiology, Semmelweis University (Budapest, Hungary).

### 2.5 Statistical analysis

Continuous variables are expressed as median ± interquartile regions (IQR) with minimum–maximum ranges, categorical variables are expressed as absolute numbers and percentages relative to their respective groups (n, %). The Wilcoxon Rank Sum test for Chao1 Alpha diversity, and PERMANOVA analysis for Bray-Curtis PCoA Beta diversity was used for statistical testing between cohorts of blood samples, by applying the statistical analysis support of CosmosID bioinformatics platform. Statistical significance was decided upon at a two-tailed *p* value of ≤0.05.

## 3 Results

### 3.1 Baseline and clinical characteristics of septic patients

Altogether, 13 septic patients were enrolled in the study. Baseline characteristics are reported in **Table 1**. The median age of the cohort was 70 ± 6 (59–83) years, 53.8% (7/13) of patients were females. Prevalent comorbidities were chronic heart disease (5/13, 38.5%) and atherosclerosis (4/13, 30.8%). At inclusion, sepsis and septic shock was diagnosed in 6/13 (46.2%) and 7/13 (53.8%) patients, respectively. All patients had an identified source, most frequently with an intraabdominal septic focus (7/13, 53.8%), and surgical or radio-interventional source control was sought in each case when clinically feasible. For comparison, baseline characteristics of the 13 non-septic control patients are reported in **Supplementary Table 1**.

**Table 1:** Baseline characteristics of septic patients included in the study. Septic sources were regrouped according to their gross anatomical regions under the total count.

Patient No.	Age(years)	Gender	Comorbidites	Symptom duration before hospital admission (days)	Sepsis severity	Septic source	Blood WBC count (G/L)	Blood ANC(G/L)	Blood ALC(G/L)	Serum CRP(mg/L)	Serum PCT (µg/L)
*1*	83	M	HT, ASO, CHD	3	Septic shock	Acute cholangitis	28.2	25.3	0.9	156	238.0
*2*	73	F	Psoriasis	5	Sepsis	Bullosus erysipelas	19.4	13.5	1.0	424	3.8
*3*	76	F	HT, ASO, CHD, mammary carcinoma	3	Sepsis	Acute gastro- enterocolitis	7.4	5.9	0.8	386	18.4
*4*	71	F	HT, ASO, CHD, CRD	2	Septic shock	Acute diverticulitis	27.3	25.8	0.2	367	11.8
*5*	70	M	HT, M. Basedow	2	Sepsis	Acute cholangitis	17.2	13.8	2.0	91	0.4
*6*	72	F	HT, RA, M. Hashimoto	1	Septic shock	Toxic shock syndrome	3.4	3.2	0.0	205	17.2
*7*	69	M	HT, ASO, CHD, prostate carcinoma	4	Septic shock	Pancolitis	24.7	21.5	1.4	325	5.8
*8*	59	M	–	5	Sepsis	CAP	14.1	13.2	1.1	235	0.3
*9*	67	F	M. Parkinson, mammary carcinoma	2	Sepsis	Acute diverticulitis	17.3	15.1	1.4	351	2.6
*10*	77	M	HT, CHD, GERD	8	Septic shock	Infective endocarditis	12.8	12.6	0.2	179	46.0
*11*	67	M	Homelessness, alcohol dependence	4	Septic shock	Fournier’s gangrene	21.7	19.0	1.3	289	3.6
*12*	66	F	M. Crohn	2	Sepsis	Acute gastro- enterocolitis	8.2	6.8	1.3	432	8.8
*13*	68	F	HT, CHD, T2DM, M. Hashimoto	1	Septic shock	UTI	6.4	5.7	0.4	158	3.4
TOTAL (n=13)	70 ± 6 (59–83)	M: 6 (46.2) F: 7 (53.8)	Alcohol dependence: 1 (7.7) ASO: 4 (30.8) CHD: 5 (38.5) CRD: 1 (7.7) GERD: 1 (7.7) Homelessness: 1 (7.7) HT: 8 (61.5) Mammary carcinoma: 2 (15.4) M. Basedow: 1 (7.7) M. Crohn: 1 (7.7) M. Hashimoto: 2 (15.4) M. Parkinson: 1 (7.7) Prostate carcinoma: 1 (7.7) Psoriasis: 1 (7.7) RA: 1 (7.7) T2DM: 1 (7.7)	3 ± 2 (1–8)	Sepsis: 6 (46.2) Septic shock: 7 (53.8)	Thoracic: 2 (15.4) IAI: 7 (53.8) SSTI: 1 (7.7) Urogenital: 2 (15.4) Other: 1 (7.7)	17.2 ± 13.5 (3.4–28.2)	13.5 ± 12.2 (3.2–25.8)	1.0 ± 0.9 (0–2.0)	289 ± 188 (91–432)	5.8 ± 13.8 (0.3–238.0)

ANC: absolute neutrophil granulocyte count, ALC: absolute lymphocyte count, ASO: atherosclerosis, CAP: community-acquired pneumonia, CHD: chronic heart disease, CRD: chronic renal disease, CRP: C-reactive protein, F: female, GERD: gastro-oesophageal reflux disease, HT: essential hypertension, IAI: intra-abdominal infection, M: male, PCT: procalcitonin, T2DM: type 2 diabetes mellitus, RA: rheumatoid arthritis, SSTI: skin and soft tissue infection, UTI: urinary tract infection, WBC: white blood cell.

Microbiological characteristics, antimicrobial therapies and outcomes of septic patients are reported in **Table 2**. Blood cultures proved to be negative in 53.8% (7/13), while *E. coli* was isolated in 30.8% (4/13) of cases. Fungaemia was not detected. Non-culture based methods documented further etiologies (*Clostridioides diffcile* and *Legionella pneumophila*) in 2/7 (28.6%) of blood culture negative cases. Seven antimicrobials were initiated empirically, with ceftriaxone (9/13, 69.2%) and metronidazole (4/13, 30.8%) is most cases. At 28 days post-admission, all-cause mortality was 15.4% (2/13), while the need for ICU admission was 30.8% (4/13).

**Table 2:** Microbiological characteristics, antimicrobial therapies and outcomes of septic patients included in the study.

Patient No.	Species isolated from blood cultures	Species proven by non-culture based methods	Antimicrobial therapy			Survival	LOS(days)	ICU admission	ICU LOS(days)
			*Empirical*	*Targeted*	*Duration*				
*1*	*Escherichia coli*	n.a.	CRO+MTZ	CRO	7	Yes	10	No	n.a.
*2*	Negative	n.a.	FAZ	n.a.	14	Yes	20	No	n.a.
*3*	*Salmonella* Enteritidis	n.a.	CRO	CRO	7	Yes	8	No	n.a.
*4*	*Streptococcus anginosus* *Clostridium perfringens*	n.a.	CRO+MTZ	CRO+MTZ	7	No	7	Yes	5
*5*	Negative	n.a.	CRO	n.a.	7	Yes	10	No	n.a.
*6*	Negative	n.a.	FAZ+CLN	n.a.	14	Yes	20	No	n.a.
*7*	Negative	Stool *Clostridioides difficile* toxin test positive	VAN	VAN	10	Yes	10	No	n.a.
*8*	Negative	Urinary *Legionella pneumophila* antigen test positive	CRO+AZI	AZI	8	Yes	11	No	n.a.
*9*	Negative	n.a.	CRO+MTZ	n.a.	5	Yes	10	No	n.a.
*10*	*Escherichia coli*	n.a.	CRO+MTZ	CRO	35	Yes	27	Yes	10
*11*	*Escherichia coli*	n.a.	TZP	CRO	7	No	8	Yes	4
*12*	Negative	n.a.	CRO	n.a.	4	Yes	8	Yes	6
*13*	*Escherichia coli*	n.a.	CRO	CRO	7	Yes	12	No	
TOTAL (n=13)	Negative: 7 (53.8) *Escherichia coli*: 4 (30.8) Other: 2 (15.4)	Positive: 2 (15.4) n.a.: 11 (84.6)	AZI: 1 (7.7) CLN: 1 (7.7) CRO: 9 (69.2) FAZ: 1 (7.7) MTZ: 4 (30.8) TZP: 1 (7.7) VAN: 1 (7.7)	AZI: 1 (7.7) CRO: 6 (46.2) MTZ: 1 (7.7) n.a.: 5 (38.5) VAN: 1 (7.7)	7 ± 3 (4–35)	Yes: 11 (84.6) No: 2 (15.4)	10 ± 4 (7–27)	Yes: 4 (30.8) No: 9 (69.2)	6 ± 2 (4–10)

AZI, azithromycin; CLN, clindamycin; CRO, ceftriaxone; FAZ, cefazolin; ICU, intensive care unit; LOS, lenght of hospital stay; MTZ, metronidazol; n.a., not applicable; TZP, piperacillin/tazobactam; VAN, vancomycin.

### 3.2 Microbiota analysis of septic and non-septic control blood samples

#### 3.2.1 Results of DNA recovery from samples

The median of isolated DNA from blood samples was 24.72 ± 18.31 (13.11–37.24) ng/µL containing human and microbial DNA. After 16S rRNA PCR, a median DNA amount of 3.61 ± 0.65 (3.22–4.87) ng/µL, and after indexing PCR, a median DNA amount of 4.87 ± 1.78 (3.76–6.23) ng/µL was amplified. The average length of index PCR products was 664 ± 53 base pairs. A total of 15.2 million valid sequences were obtained, resulting in 10.7 million high-quality reads. The median number of reads within one sample was 169411 ± 29511 (132487–195364) in the control patients’ group, 206754 ± 21332 (187999–245872) in the septic patients’ subgroup and 335678 ± 23521 (319442–351263) in the septic shock patients’ subgroup.

#### 3.2.2 Diversity indices among septic patients and non-septic controls

**Figure 1:**
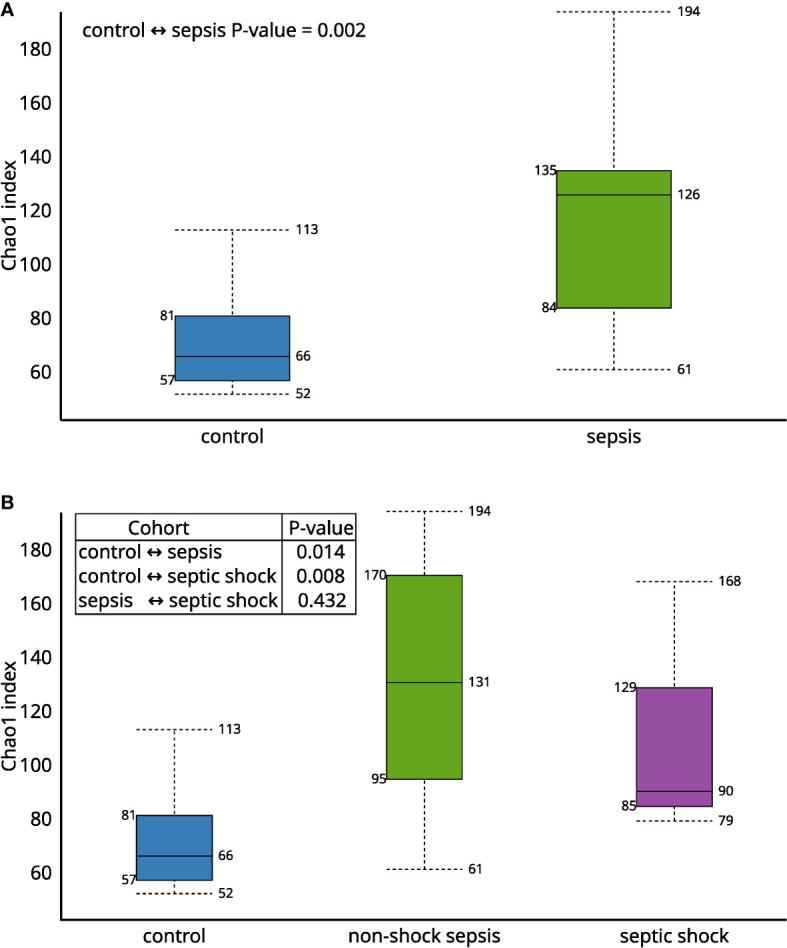
The Chao1 alfa diversity between **(A)** septic and control patients; **(B)** patients with non-shock sepsis, septic shock and control patients: The difference of alpha diversities between control/septic, control/non-shock septic and control/septic shock patients are significant. There is no significant difference between the two septic subgroups.

The Chao1 alfa diversity was higher in the septic group, compared to the control group in a statistically significant manner (**Figures 1A, B**). By further subgrouping within the septic group, the alpha diversity of both the non-shock and septic shock subgroups is significantly higher than the alpha diversity of the control group. However, there was no statistically significant difference detected in the alpha diversity of the two septic subgroups.

**Figure 2:**
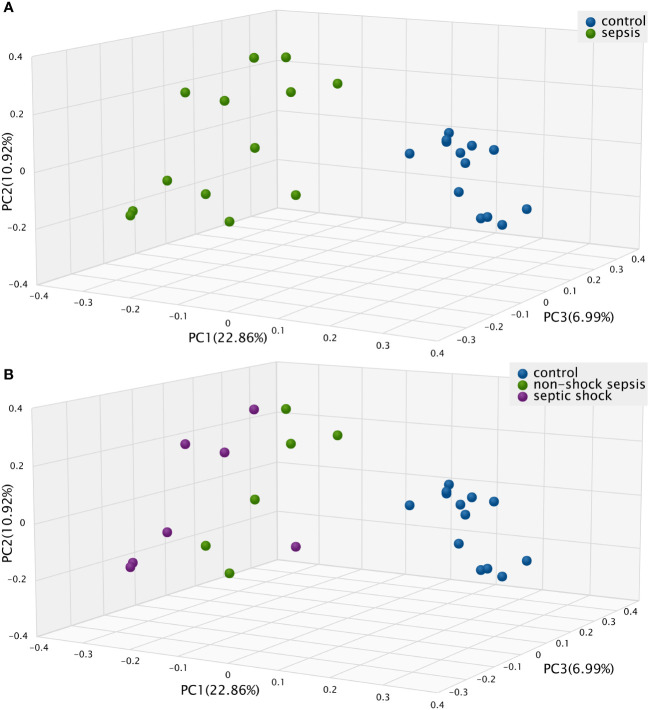
The *Bray-Curtis* Principal Component Analysis (PCoA) Beta diversity between **(A)** septic and control patients; **(B)** patients with non-shock sepsis, septic shock and control patients: The difference of beta diversities between control/septic, control/non-shock septic and control/septic shock patients are significant. There is no significant difference between the two septic subgroups.

The Bray-Curtis beta diversity Principal Component Analysis (PCoA) shows significantly distinct clusters among septic vs. non-septic patients (**Figure 2A**), however, although there is a trend in the shift of septic subgroups compared to the control group, the two subgroups do not form new clusters that are significantly different from each other (**Figure 2B**).

#### 3.2.3 Differential abundance among septic patients and non-septic controls

**Figure 3:**
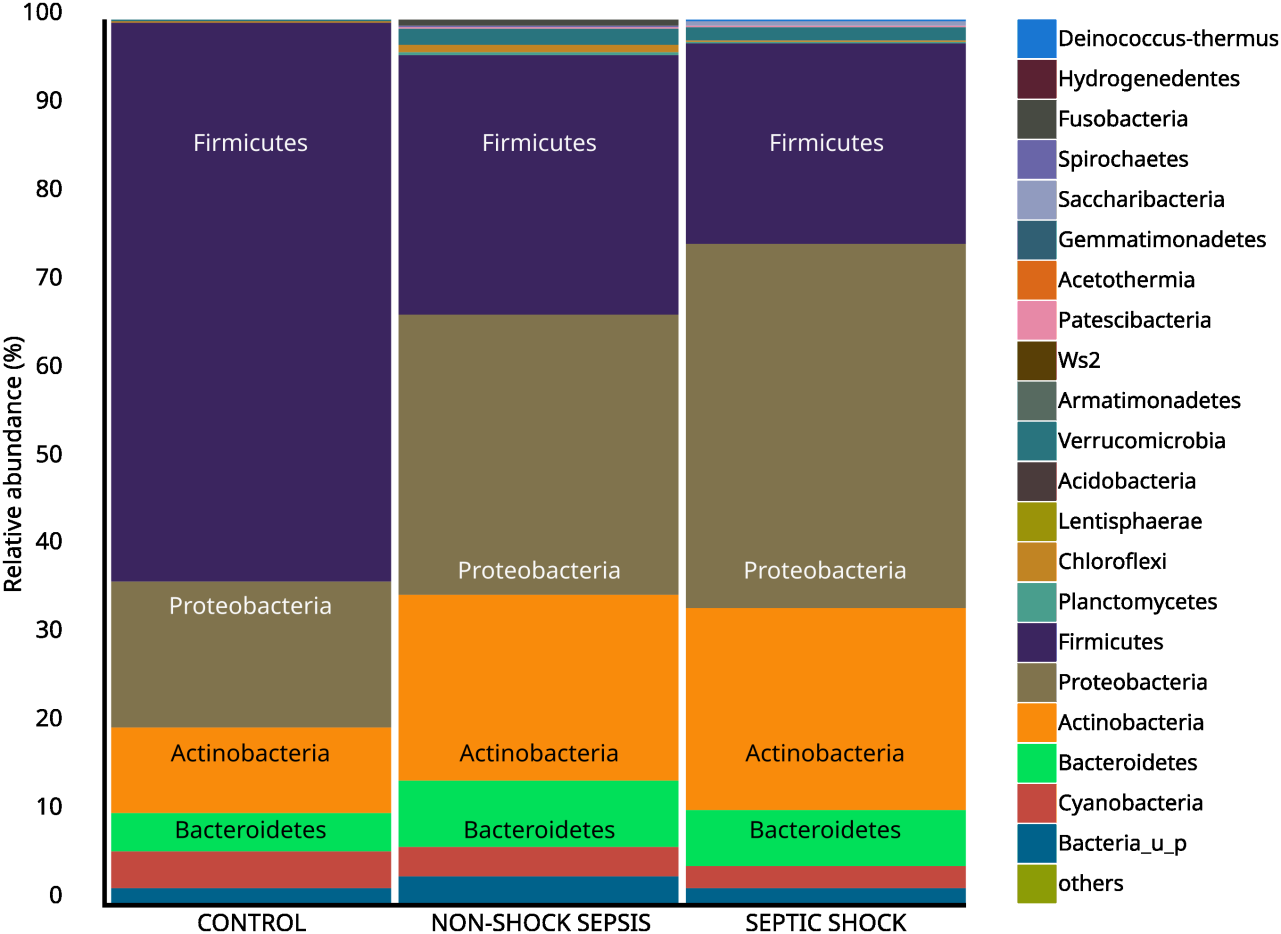
Abundance of taxa at the phylum level among non-septic control, non-shock sepsis and septic shock patients.

The comparison of the values of the septic and non-septic control groups resulted in a significant difference in the abundance of phyla *Firmicutes* (25.7% vs. 63.1%; p<0.01), *Proteobacteria* (36.9% vs. 16.6%; p<0.01) and *Actinobacteria* (22.1% vs. 9.9%; p<0.01) (**Figure 3**). Furthermore, phylum abundance differences showed a gradual decrease among *Firmicutes*, and an increasing trend among *Proteobacteria* in the control, non-shock sepsis and septic shock groups, respectively

**Figure 4:**
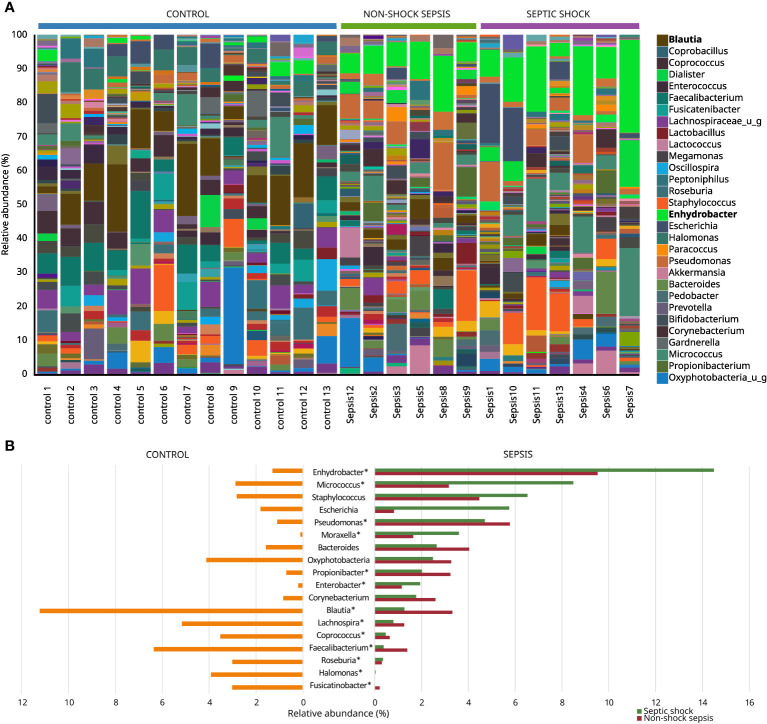
Most abundant genera among non-septic control, non-shock sepsis and septic shock patients **(A)**, and genera with the highest overall relative abundance among non-septic control and septic patients **(B)**. * means significant difference.

Only six of thirteen traditional blood culturing resulted in positive isolates which partially correlated with microbiota abundance results. **Table 3**. showed *Escherichia coli* as the most prevalent isolated etiopathogen (4/7, 57.1%) of blood culture samples, however, the highest abundance of *Escherichia coli* was only obtained during 2/4 (50.0%) microbiota analyzes from related blood samples. Furthermore, in septic patients, the 3 most abundant genera belonged mainly to the *Proteobacteria* phylum in microbiota analysis. Bacteria isolated from blood cultures also appeared in the microbiota analysis for each patient, but their abundance varied on a wide scale from 0.11% to 17.43%. *Salmonella* Enteritidis and *Clostridium perfringens*, which were interpreted as etiopathogens based on blood culture results, appeared only in the microbiota analysis of septic patients. In contrast, *Streptococcus anginosus* and *Escherichia coli* taxa were present with varying abundance in the control and septic groups, but these differences were not statistically significant. Surprisingly, contrary to positive non-culture based results of urinary and stool tests, *Clostridioides difficile* and *Legionella pneumophila* taxa were not detected during blood microbiota analysis of the affected patients. Comparing the results of **Table 3** and **Figure 4A**, bacteria interpreted as etiopathogens based on cultured or non-cultured routine laboratory results were confirmed to be the most frequently present only in Sepsis 1 and Sepsis 10 patients by microbiome analysis.

**Table 3:** Results of species isolated from blood cultures and abundance during microbiota analysis in septic patients.

Patient No.	Species isolated from blood cultures	Abundance of cultured species in the microbiota	First most abundant genus in the microbiota	Second most abundant genus in the microbiota	Third most abundant genus in the microbiota
*1*	*Escherichia coli*	17.43%	Escherichia 17.43%	Pseudomonas 11.68%	Enhydrobacter 7.97%
*2*	–	–	Enhydrobacter 8.16%	Micrococcus 6.52%	Enterococcus 5.60%
*3*	*Salmonella* Enteritidis	0.11%	Pedobacter 8.04%	Enhydrobacter 7.05%	Pseudomonas 6.75%
*4*	*Streptococcus anginosus*	0.21%	Enhydrobacter 20.29%	Micrococcus 10.57%	Pseudomonas 8,32%
	*Clostridium perfringens*	1.99%			
*5*	–	–	Enhydrobacter 10.96%	Akkermansia 8.41%	Blautia 5.86%
*6*	–	–	Bacteroides 10.55%	Enhydrobacter 9.34%	Akkermansia 6.84%
*7*	–	–	Enhydrobacter 27.21%	Micrococcus 20.08%	Moraxella 13.66%
*8*	–	–	Enhydrobacter 16,58%	Pseudomonas 13.97%	Faecalibacterium 5.75%
*9*	–	–	Staphylococcus 14.82%	Propionibacterium 8.65%	Lactobacillus 6.25%
*10*	*Escherichia coli*	15.89%	Escherichia coli 15.89%	Enhydrobacter 13.11%	Staphylococcus 9.42%
*11*	*Escherichia coli*	1.53%	Enhydrobacter 19.29%	Staphylococcus 15.73%	Micrococcus 12.62%
*12*	–	–	Oxyphotobacteria 14.37%	Lactococcus 8.71%	Pseudomonas 7.19%
*13*	*Escherichia coli*	5.36%	Staphylococcus 11.20%	Escherichia 5.36%	Micrococcus 5.24%

Background colors: blue: Proteobacteria, yellow: Firmicutes, green: Actinobacteria, purple: Bacteroidetes, grey: Cyanobacteria, brown: Verrucomicrobia.

The microbiota of non-septic control patients contained the *Enhydrobacter* genus in 8/13 (61.5%) cases with an abundance of 1.18−4.07%, and this taxon was also detected in 11/13 (84.6%) septic cases with a statistically significantly higher abundance of 3.92−27.21% (p<0.001). The abundances of *Micrococcus* (0.37%−20.08% vs. 0.00%−11.64%; p=0.03) and *Pseudomonas* (0.59%−13.97% vs. 0.17%−2.55%; p=0.04) genera were also statistically significantly higher in the sepsis group, compared to non-septic controls. Although with low abundance values, the genera *Moraxella, Propionibacterium* and *Enterobacter* also occurred in significantly higher quantities in the sepsis group, while the abundance of the genera *Corynebacterium, Bacteroides* and *Staphylococcus* did not show any statistically significant difference. The genera with high abundance in the control group, such as *Blautia, Faecalibacterium, Coprococcus*, *Lachnospira, Roseburia, Halomonas* and *Fusicatinobacter* were all present in significantly higher amounts in this group, compared to septic patients (**Figures 4A, B**).

## 4 Discussion

### 4.1 Main findings of the present study

In our prospective observational study, whole blood samples originated from 13 hospitalized patients with community-acquired sepsis were examined with traditional blood culturing, in parallel to 16S rRNA microbiota analysis. To our knowledge, this is the first study evaluating the composition and changes of blood microbiota in adult patients with community-acquired sepsis, with correlation to non-septic control patients.

We found that Chao1 alpha diversity index showed a significant difference and the Bray-Curtis beta diversity revealed 2 significantly separate clusters between septic and control patients. We also found that relative microbiota differences between patients were more pronounced at the phylum level, mainly by *Firmicutes* (25.7% vs. 63.1%; p<0.01) and *Proteobacteria* (36.9% vs. 16.6%; p<0.01). Interestingly, the most prevalent etiopathogen isolated from blood cultures was *Escherichia coli*, from mainly intraabdominal septic sources, but only six of thirteen traditional blood culturing resulted in positive isolates correlated with microbiota abundance results.

Moreover, a relative scarcity of *Faecalibacterium*, *Blautia, Coprococcus* and *Roseburia* genera, with an abundance of *Enhydrobacter*, *Pseudomonas* and *Micrococcus* genera was observed among septic patients. *Faecalibacterium* genus has a sole known species, *Faecalibacterium prausnitzii*, a Gram positive anaerobic rod with a high abundance in the human colon. By fermenting dietary fibers to butyrate and other short-chain fatty acids, the “eubiotic” presence of *F. prausnitzii* in the gut homeostasis might translate to anti-inflammatory properties, such as improvement of the intestinal colonization resistance and mucosal permeability. In contrast, decreased abundance of *F. prausnitzii* has been associated with inflammatory bowel diseases and colorectal cancer. Similar to *Faecalibacterium* genus, members of the *Blautia* (21 identified species), *Roseburia* (10 identified species) and *Coprococcus* (5 identified species) genera are also butyrate-producing Gram positive anaerobic bacteria, most abundant in the human feces (Machiels et al., 2014; Martin et al., 2018). The present study did not analyze the change in the gut microbiome of sepsis, but it could be noted that the abundance decrement in the 4 taxa in the blood of our patients might partially be related to a similar microbiome change in the gut, as this notion was already proven by others (Du et al., 2021; Yu et al., 2022). We might hypothesize that the decreased amount of these “eubiotic” genera could be biomarkers of intestinal dysfunction during sepsis, and their relative scarcity may indicate an ongoing facilitation of systemic inflammation and invasion of other microbes to deeper tissues and the bloodstream, but this idea has yet to be proven. *Enhydrobacter* genus with its one identified species, *Enhydrobacter aerosaccus*, a Gram negative facultative anaerobic rod, was found to be abundant in blood samples of most patients with sepsis. As *Enhydrobacter aerosaccus* was first isolated from an eutrophic lake in 1987, it may be surprising to identify this bacterium in several cases. However, there are increasing amount of data detailing *Enhydrobacter aerosaccus* as a possible member of the human skin microbiota. Profiled by age, the composition of the skin microbiome confirmed an increased presence of *Enhydrobacter* at older ages (Kim et al., 2021). A positive correlation was demonstrated between the vulnerability of the skin barrier and the abundance of *Enhydrobacter* genus (Zhou et al., 2022). Information exists about this taxon not only as a component of the skin microbiota, but the increasing level of *Enhydrobacter* genus in blood microbiota was proved to be associated with cardiovascular disease mortality (Lawrence et al., 2022). Knowing these correlations, it is not surprising that the blood microbiota of our septic and septic shock patients belonging to the older age group contained an increasing amount of the *Enhydrobacter* genus compared to the blood samples of healthy controls.

### 4.2 Previous studies from the literature

The notion that the human blood is physiologically not sterile goes back to the 1960s, when the presence of viable bacteria was first documented in blood specimens of healthy individuals. Since then, numerous studies have aimed to prove this hypothesis by examining different clinical preparations of blood samples with several culture and non-culture based microbiological methods, including radioactive nucleoside and amino acid uptake (outdated), classical culturing of whole and filtrated blood, fluorescence *in situ* hybridization (FISH) probing, and most recently, PCR based amplification and sequencing of different bacterial DNA and RNA targets, including 16S rRNA genes and cell-free DNA (Castillo et al., 2019)

Interpretation of blood microbiota data is somewhat conflicting. It should be noted that the type of specimen may influence the diversity and abundance profiles found in the blood, creating a microbiologically relevant difference between results of each study. The types of blood specimens could be grouped as whole blood, cellular components such as leukocytes, erythrocytes, the buffy coat, mononuclear cells and neutrophil granulocytes, and non-cellular components, such as serum, plasma, and extracellular vesicles. However, it is generally thought that analysis of whole blood might be the most representative specimen for blood microbiota, since it consists of all blood elements (Suparan et al., 2022). According to published data, the most abundant phyla of whole blood “eubiotic” microbiota was *Proteobacteria*, followed by *Firmicutes* and *Actinobacteria*, but results are highly discordant on the genera level, identifying relevant microbiological variability, probably originating from oral and skin communities (Paisse et al., 2016; Whittle et al., 2018) Lastly, comparative data showed that factors affecting the profiles of blood microbiota might be age, gender and geographic location (D’Aquila et al., 2021; Panaiotov et al., 2021).

Most previous literature data about the blood microbiota during sepsis are derived from studies enrolling patients from the healthcare setting, including potential sources of nosocomial acquisition. In one of the first observational studies reporting on the blood microbiota changes during sepsis, *Gosiewski et al.* collected whole blood from 23 healthy volunteers and 62 septic patients recently undergoing cardiothoracis surgical procedures, and analyzed samples by 16S rRNA metagenomic next-generation sequencing. They reported that all blood samples contained bacterial DNA at detectable levels, and a higher diversity and bacterial predominance of the order *Bifidobacteriales* was proven among healthy individuals. Moreover, the abundance of the phyla *Proteobacteria* decreased in blood samples from healthy subjects, while the phyla *Actinobacteria* decreased in septic patients (Gosiewski et al., 2017).

Other researchers sought to assess the possible origins of blood microbiota in septic patients among the microbiological changes which characterize this group. *Wang et al.* recruited 204 cases with recent surgical intervention, and collected blood samples for high‐throughput DNA sequencing to detect whole blood and neutrophil-specific microbiota. The authors found that both whole blood and neutrophil-derived microbiota of surgical patients reflected diverse composition, with an abundance of the phyla *Proteobacteria, Firmicutes* and *Bacteroidetes*, and the origin of approximately 80% of blood bacteriome could be traced back to the gut microbiome Furthermore, the presence of *Agrococcus, Polynucleobacter* and *Acidovorax* in the blood positively correlated with sepsis-related organ dysfunction and serum lactate levels. The authors therefore concluded that the dysbiotic state of blood microbiota among surgical patients with sepsis might partially originate from the gut microbiome, and *Agrococcus* may play a role in the clinical progression of sepsis (Wang et al., 2021).

These findings were also mirrored in recent reviews, pointing out that the frequent loss of “eubiotic” *Bacteroidetes* and *Firmicutes*, and novel abundance of *Proteobacteria* could be documented after the onset of sepsis. Moreover, the disproportionate shift from the dominance of *Faecalibacterium* and other commensals may facilitate the colonization of the intestinal microbiome by healthcare-associated pathogens during sepsis (Adelman et al., 2020; Miller et al., 2021).

Also, it has been proposed that the intestinal microbiome and its derived metabolic products are a relevant modulator of the host immune response, thereby promoting homeostasis and interfering with systemic infection. The lack of an overwhelming host immune response generated against members of the circulating blood microbiota of non-septic, healthy patients might partially be explainable by central and peripheral immune tolerance directed against these “eubiotic” components with low virulence, probably induced by a cross-talk of the gut microbiome, but this phenomenon needs further research clarification (Niu and Chen, 2021).

### 4.3 Limitations of the study

Our study has limitations. Firstly, the relatively low number of enrolled septic patients calls for an overall cautious interpretation of our results. Second, a near-perfect matching of an adequate control group might be challenging, as the blood microbiota may become altered by chronic comorbidities to show an already “dysbiotic” state. Third, the time between potential exposure to infectious agents and diagnosis of sepsis was not unambiguously explorable for some patients. Lastly, undetected blood sampling or handling errors might have biased our final microbiological results.

## 5 Conclusion

In conclusion, adult patients hospitalized with community-acquired sepsis may harbor specific “dysbiotic” changes in the taxonomic composition and abundance of blood microbiota, compared to non-septic control patients. Furthermore, traditional blood culturing results might only partially correlate with results from 16S rRNA metagenome sequencing. More studies are possibly warranted to shed light on the relevance of these phenomena.

## Data availability statement

The microbiological datasets generated during the current study are available in the Short Read Archive (SRA) of National Center for Biotechnology Information (NCBI) under accession number: PRJNA886752 (free available from 06.12.2022.). Anonymized clinical data of patients are available from the corresponding author on reasonable request.

## Ethics statement

The studies involving human participants were reviewed and approved by Institutional Review Board of Semmelweis University (Permission Number: SE RKEB: 87/2019) South Pest Central Hospital, National Institute of Hematology and Infectious Diseases (Permission Number: ESZSZK EB 37/2016). The patients/participants provided their written informed consent to participate in this study.

## Author contributions

Conceptualization, BS, RK, DS, and EO. Data curation, BS, RK, and EO. Funding acquisition, BS and DS. Investigation, NM, KP, EV, KK, and EO. Methodology, BS, RK, EO, NM, and KP. Project administration, BS, RK, and EO. Resources, BS, RK, EV, KK, EO, and DS. Software, EO and DS. Supervision, BS and EO. Validation, BS, RK, EV, KK, EO, and DS. Visualization, EO. Writing – original draft, BS, RK, and EO. Writing – review and editing, BS, RK, DS, and EO. All authors read and approved the final manuscript. All authors agree to be accountable for the content of the work.
