# Supplementary material for: Composition and changes of blood microbiota in adult patients with community-acquired sepsis: A pilot study from bench to bedside
Source: Front Cell Infect Microbiol. 2022 Dec 13;12:1067476. doi: 10.3389/fcimb.2022.1067476 (PMC9794134; doi:10.3389/fcimb.2022.1067476)
Supplement: Supplementary file 1 [file Table_1.docx]

| **Patient No.** | **Age (years)** | **Gender (n, %)** | **Comorbidites (n, %)** |
| --- | --- | --- | --- |
| *1* | 86 | F | HT, ASO |
| *2* | 89 | M | HT, ASO, GERD |
| *3* | 71 | F | T2DM, M. Hashimoto |
| *4* | 68 | F | T2DM, M. Basedow |
| *5* | 68 | M | HT, CHD, BPH |
| *6* | 76 | F | HT, ASO, CRD |
| *7* | 69 | M | HT, ASO, urothelial carcinoma |
| *8* | 84 | M | BPH, glaucoma |
| *9* | 70 | F | HT, ASO |
| *10* | 64 | F | T2DM, M. Hashimoto |
| *11* | 79 | M | HT, ASO, CHD |
| *12* | 72 | M | – |
| *13* | 72 | F | RA |
| TOTAL  (n=13) | 72±10  (64–89) | M: 6 (46.2)  F: 7 (53.8) | ASO: 6 (46.2)  BPH: 2 (15.4)  CHD: 2 (15.4)  CRD: 1 (7.7)  GERD: 1 (7.7)  Glaucoma: 1 (7.7)  HT: 7 (53.8)  M. Basedow: 1 (7.7)  M. Hashimoto: 2 (15.4)  RA: 1 (7.7)  T2DM: 3 (23.1)  Urothelial carcinoma: 1 (7.7) |

ASO: atherosclerosis, BPH: benign prostatic hyperplasia, CHD: chronic heart disease, CRD: chronic renal disease, GERD: gastro-oesophageal reflux disease, HT: essential hypertension, F: female, M: male, T2DM: type 2 diabetes mellitus, RA: rheumatoid arthritis

**Supplementary Table 1.** Baseline characteristics of non-septic control patients included in the study.
